# Spatial and temporal evolution of natural and anthropogenic dust events over northern China

**DOI:** 10.1038/s41598-018-20382-5

**Published:** 2018-02-01

**Authors:** Xin Wang, Jun Liu, Huizheng Che, Fei Ji, Jingjing Liu

**Affiliations:** 1 Key Laboratory for Semi-Arid Climate Change of the Ministry of Education, College of Atmospheric Sciences, Lanzhou University, Lanzhou, 730000 China; 2 Key Laboratory for Atmospheric Chemistry, Chinese Academy of Meteorological Sciences, CMA, Beijing, 100081 China; 3 School of Mechanical and Precision Instrument Engineering, Xi’an University of Technology, Xi’an, 710048 China

## Abstract

Mineral dust interacts with radiation and cloud microphysics in East Asia can affect local and regional climate. In this study, we found that the occurrences of dust storms, blowing dust, and floating dust over northern China has decreased 76.7%, 68.5%, and 64.5% considerably since the beginning of this century. Based on a multi-dimensional ensemble empirical mode decomposition (MEEMD) method, a steady decrease in zonal maximum wind speed (up to −0.95 m/s) in the Northern Hemisphere was largely responsible for this recent decline in dust event occurrences. Then, a new detection technique that combines multi-satellite datasets with surface observations of dust events is developed to estimate the contribution of anthropogenic dust column burden from disturbed soils to the observed total dust. It is found that the percentage of the anthropogenic dust column burdens to total mineral dust is up to 76.8% by human activities during 2007–2014 in eastern China, but only less than 9.2% near desert source regions in northwestern China. However, we note that the anthropogenic effects on the dust loading for both regions are non-negligible.

## Introduction

The Taklimakan, Gobi, and other deserts (e.g., the Badain Jaran and Tengger deserts) in central Asia are major sources of mineral dust^1–3^. From late winter to spring, large quantities of natural mineral dust generated during dust outbreak events (i.e., dust storms) can be transported eastward by the prevailing westerlies across East China, Japan, the northwest Pacific Ocean, and even North America^4–6^. Mineral dust particles can affect local and regional weather and climate by absorbing and scattering solar radiation, interacting with the biosphere, and modifying cloud microphysical properties and precipitation efficiency; thus, they can change large-scale atmospheric conditions through their atmospheric warming effects and surface cooling effects^7–13^.

Northeastern China and the surrounding regions are generally regarded as industrial areas that have been severely affected by human activities^3,14–16^. Because estimates of dust emissions from disturbed soils are poorly constrained, we define mineral dust from areas disturbed by human activities, such as deforestation, overgrazing, and agricultural and industrial activities, as anthropogenic dust^17–23^. It is, however, challenging to distinguish anthropogenic dust from natural dust. Due to differences in the sensitivity to visible and near-infrared radiation, anthropogenic dust over oceans or lakes is characterized by a magenta color in satellite images, while natural dust appears purplish-yellow^24^. Based on direct satellite measurements combined with land use datasets, mineral dust due to human activities has been estimated to account for about 25% of total dust aerosols globally^25^. Similarly, mineral dust from agricultural areas has been estimated to contribute <10% to the global dust loading^20^. A recent study using multi-satellite measurements and planetary boundary layer height retrievals found that anthropogenic dust contributions to regional emissions are as high as 91.8% in eastern China and 76.1% in India^26^. Although several attempts have been made to investigate the climate effects of anthropogenic dust, large uncertainties remain in the estimated contribution of anthropogenic dust that are largely based on multi-satellite retrievals^27,28^. For instance, current estimates of the anthropogenic dust as a percentage of global dust emissions range from less than 10% to a maximum of 50%^20,29^. Such a large range reflects the current limited ability by satellite remote sensing to effectively assess the optical properties, chemical compositions, and source regions of anthropogenic dust^30^.

Previous studies have investigated the long-term trends in the occurrence frequency of dust events based on surface observations of wind speed, visibility, and dust events in East Asia^26–28^; however, many of these studies examined the variations in dust event occurrences mainly based on station observations before 2000. To help understand historical and future changes in natural and anthropogenic dust, the contribution of dust emissions from regions with disturbed soils to atmospheric dust loading needs to be quantified accurately. In this study, we first examine the historical changes in dust event occurrences in northern China since 1960 based on the multi-dimensional ensemble empirical mode decomposition (MEEMD) technique. Then, a novel method is developed based on ref.^26^ to investigate the contribution of anthropogenic dust to atmospheric dust loading in regions with disturbed soils over China during 2007–2014. In contrast to earlier studies, the new method significantly reduces the uncertainty range for the estimated column burden of anthropogenic dust by combining multiple satellite retrievals with surface observations of dust events. Details of the datasets and methods used in this study are provided in the Data and Methods section.

## Results

Here, we extend the trend analyses of daily dust event occurrences associated with the maximum wind speed and wind direction to cover the period from 1960–2014 using meteorological observations provided by the National Meteorological Information Center of China. Dust event occurrences estimated at the weather stations were mapped onto a 1° × 1° latitude-longitude grid for 1960–2014, and dust products retrieved by multiple satellites for 2007–2014 are described in the Data and Methods section. Dust events have been classified into three categories as dust storms, blowing dust, and floating dust by the National Weather Bureau of China (1979)^31–34^. Dust storms is defined as the large quantities of dust particles transport in the atmosphere (Visibility < 1 km), while floating dust is defined as suspended dust derived from upwind sources (Visibility < 10 km). The definition of blowing dust is similar with floating dust, but for dust that is emitted from local sources.

**Figure 1:**
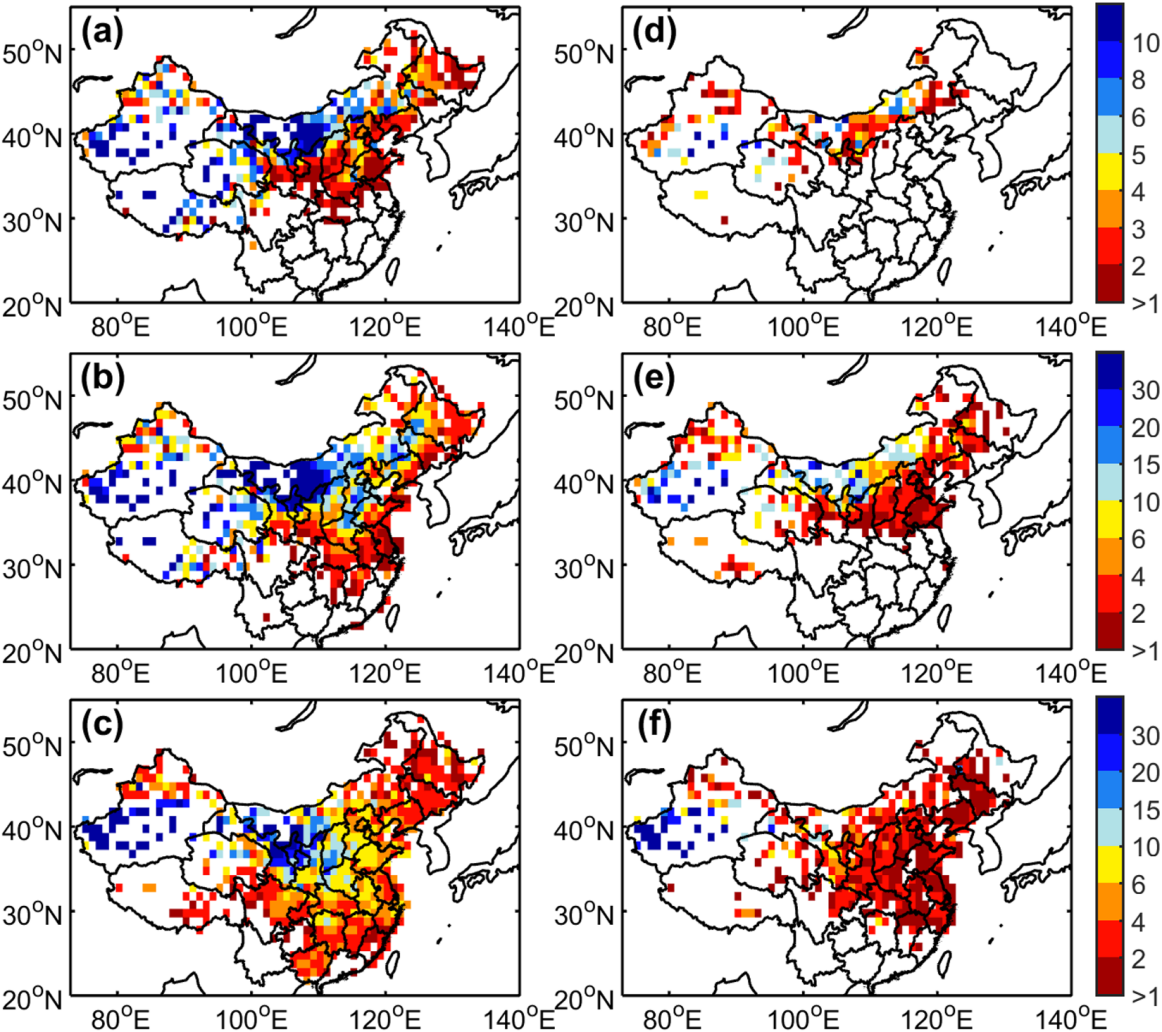
Comparison of the gridded mean frequency (in time) of annual dust events for (**a**) and (**d**) dust storms; (**b**) and (**e**) blowing dust; (**c**) and (**f**) floating dust over China during (left column) 1960–1999 and (right column) 2000–2014 observed from weather stations. The maps in the figure are generated using the MATLAB software (Version:R2016a (9.0.0.341360) & http://www.mathworks.com/products/matlab/?s_tid=srchtitle).

The occurrence frequency of dust events was considerably lower in 2000–2014 than in 1960–1999 in the arid and semi-arid regions across northern China (Fig. 1). The frequencies of dust storms, blowing dust and floating dust decreased by as much as 76.7%, 68.5%, and 64.5%, respectively, near the source regions around the Taklimakan Desert, Gobi Desert, and Badain Jaran Desert. Although previous studies have noted the negative trends in dust storms frequency up to 2004^34^, it is still surprising that the frequencies of all types of dust events decreased so substantially during 2000–2014. Additionally, among the three dust types, floating dust exhibits the largest coverage over northern China, even during 2000–2014 (Fig. 1). Blowing dust and dust storms are mainly caused by natural processes, such as strong winds^34,35^. In contrast, human activities contribute to the occurrences of floating dust in densely populated eastern China.

**Figure 2:**
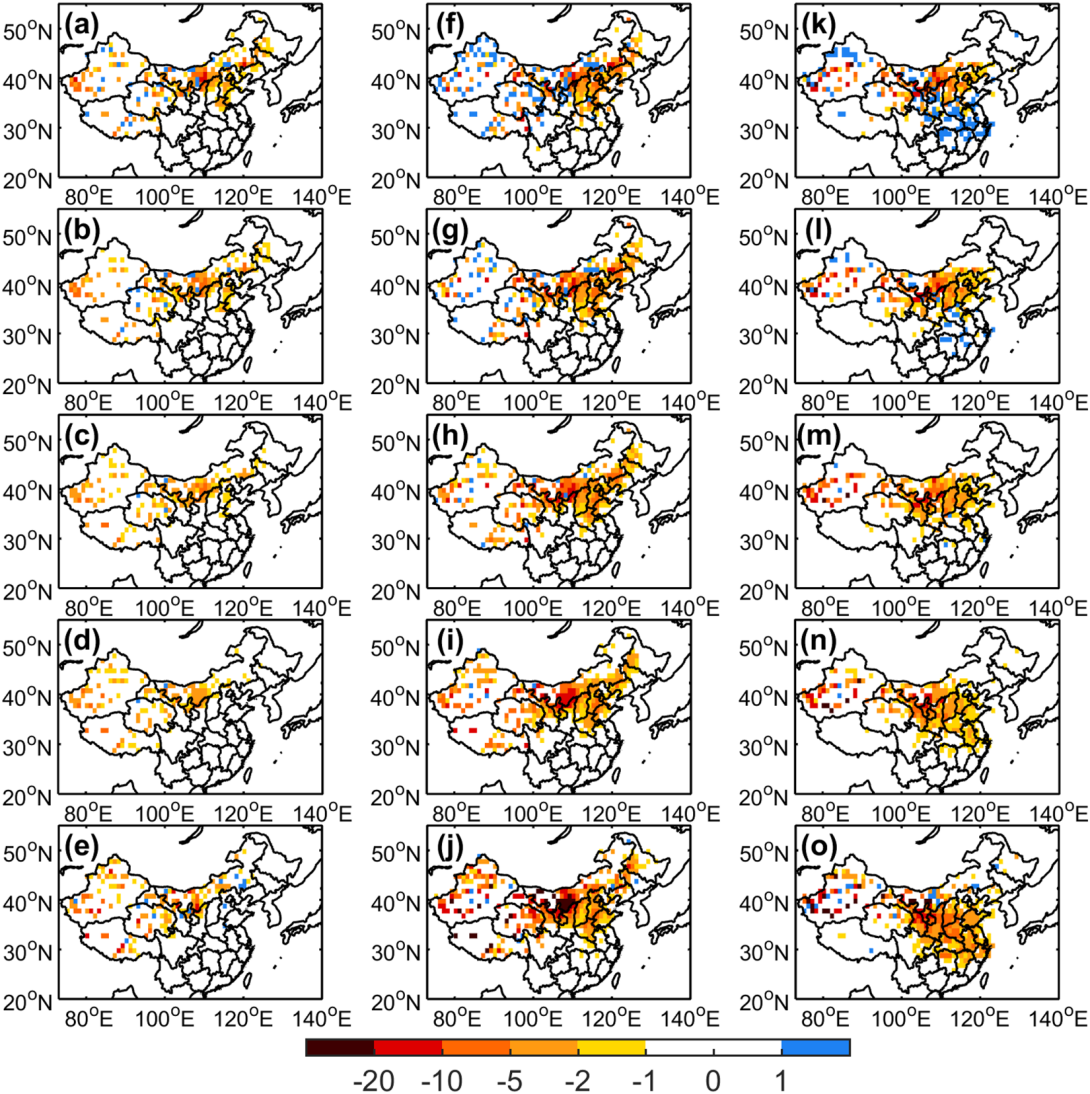
Spatial evolution of decadal changes in dust storms in the (**a**) 1960–1969, (**b**) 1970–1979, (**c**) 1980–1989, (**d**) 1990–1999, and (**e**) 2000–2014, respectively, based on MEEMD. From (**f**) to (**j**) same as from (**a**) to (**e**) but for blowing dust. From (**k**) to (**o**) same as from (**a**) to (**e**) but for floating dust. The maps in the figure are generated using the MATLAB software (Version:R2016a (9.0.0.341360) & http://www.mathworks.com/products/matlab/?s_tid=srchtitle).

To reveal the amplitudes and spatial patterns of dust-event frequencies, we preformed MEEMD analyses for a decade of change from 1960 to 2014 (Fig. 2). The results (Fig. 2a,b,f,g,k,l) show only slight decreases in dust event occurrences for all types of dust events during 1960–1979 but large declines since 1980 (Fig. 2c,d,h,i,m,n). The decadal changes in dust storms were mainly negative in the Inner Mongolia region during 1960–2014, and the blowing dust occurrences have decreased at all observational stations since 1980. Furthermore, floating dust is not only highly correlated with natural dust events but also influenced by the anthropogenic forcing due to human activities. Therefore, the magnitude of the trend for floating dust was greater than that of blowing dust in the lower latitude regions. Notably, in several regions of western China, blowing dust and floating dust switched from an increasing trend to a decreasing trend in approximately 2000 (Fig. 2f–j,k–o).

**Figure 3:**
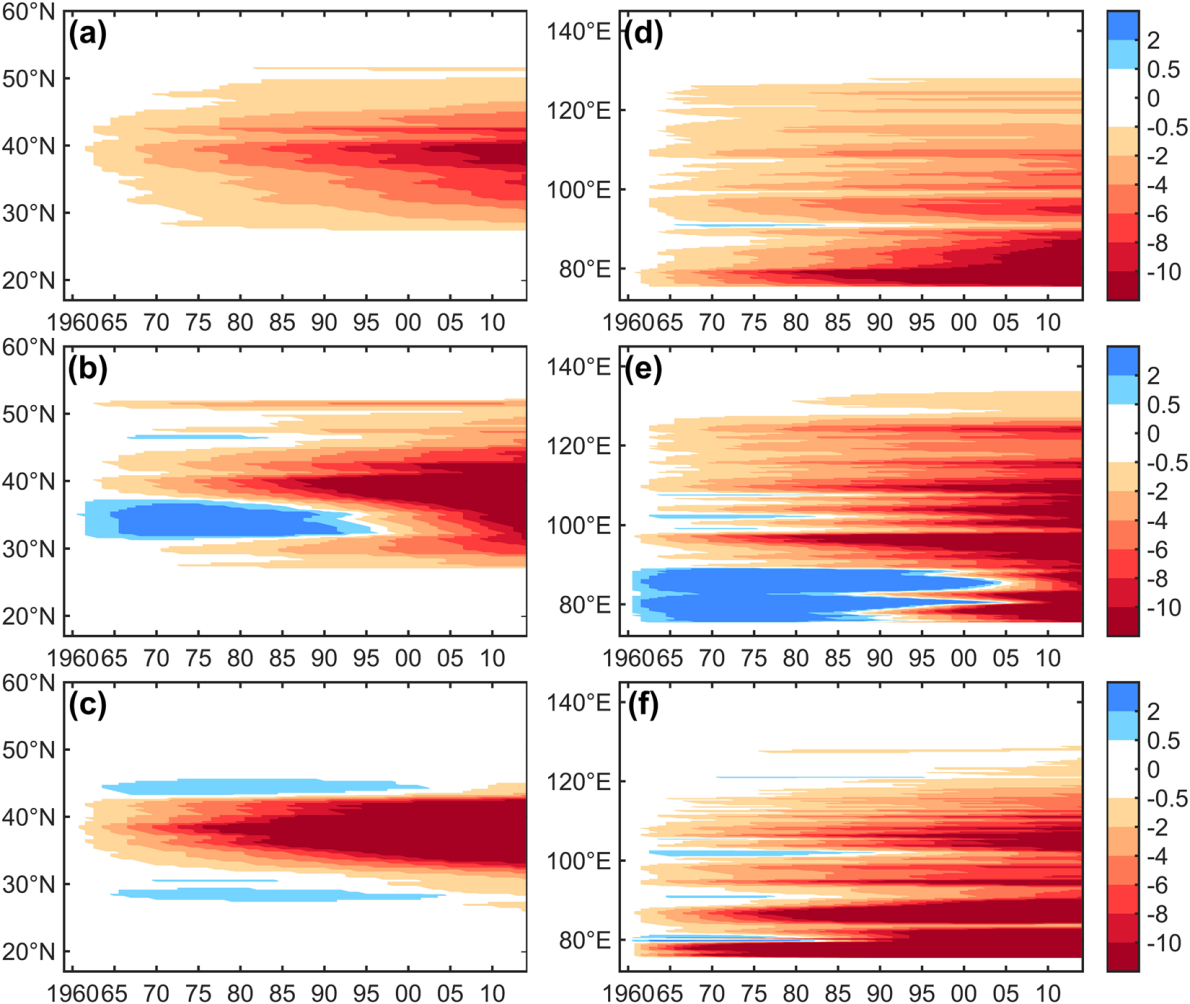
Evolution of the zonal mean frequency of (**a**) dust storms, (**b**) blowing dust, and (**c**) floating dust over China for the periods of 1960–2014. From (**d**) to (**f**) same as from (**a**) to (**c**) but for the meridional mean.

Therefore, the zonally and meridionally averaged occurrences of dust storms indeed had a single structure (Fig. 3a and d). The zonally averaged decrease (>2 days) first occurred at approximately 40 °N in 1970 and spread to lower latitudes across northern China in the 1980s. However, large differences exist between the evolution of floating/blowing dust occurrences and the evolution of dust storms occurrences. Both floating and blowing dust occurrences clearly show slight increasing trends at the beginning of the 1960s (Fig. 3b,c,e and f). Increased blowing dust events occurred in certain areas (30–40°N, 75–87°E) from 1960 to 1995, similar to the slight increase in floating dust occurrences at the edge of 30–42°N. However, notable decreasing trends in floating dust and blowing dust primarily emerged in the northern subtropical regions at the center of 38°N in northern China. The amplitude of the decreasing trend grew slowly prior to the 1980s, but the amplitude grew much faster in mid-latitude areas after the 1980s. Moreover, the −3~−21 days lines joined by approximately 1981–2000. From 2000 onwards, all types of dust events show the fastest decreasing trends at all latitudes and longitudes. The regions with the greatest decreases (−9~−32 days) after 2000 in northern China not only included the desert areas but also included the northeast and surrounding areas. However, it remains unclear whether the decreasing dust event trends are due to physical and dynamic mechanisms related to land-ocean interactions under global warming in recent decades, and not enough evidence is available to determine whether the phenomenon is associated with anthropogenic forcing (e.g., aridity) at different timescales.

**Figure 4:**
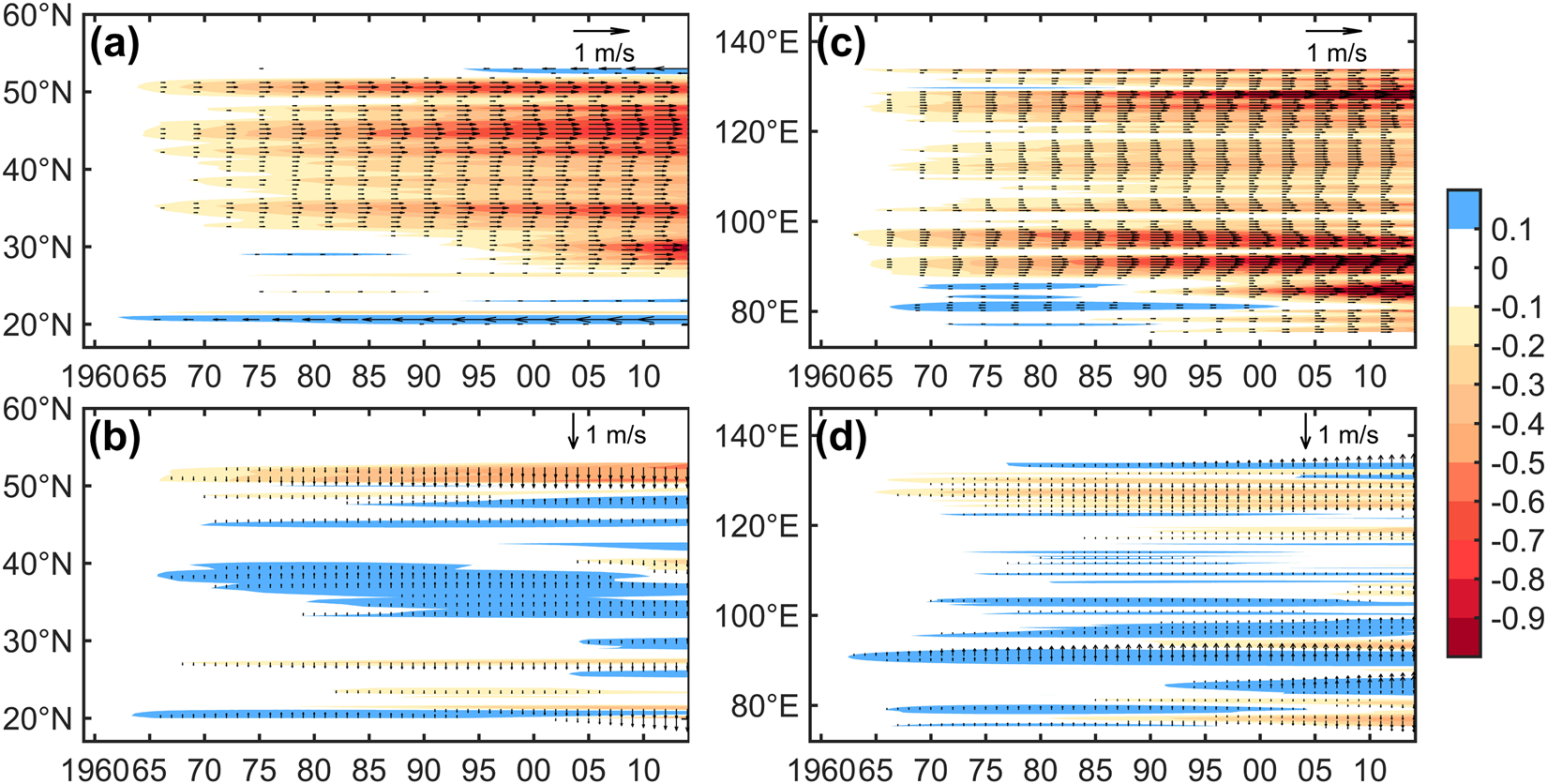
Evolution of (**a**) the zonal mean and (**c**) meridional mean of the maximum surface wind (u component from west to east). (**b**) and (**d**) same as (**a**) and (**c**) but for the maximum surface meridional wind (v component from north to south).

In this study, we divide the maximum wind speed as zonal (U) and meridional (V) components to present the wind vectors as Fig. 4 indicated. For a zonal average perspective, the trends of dust event occurrences are accompanied by a marked weakening in westerly maximum wind speed (>0.2 m/s) started over the high latitude regions (35–50° N) in China and accelerated until around 1980 (Fig. 4a). We note that the decreased dust events in northern China after the 1980s was concurrent with the weakening of the westerly jet stream and a shift in geopotential height over the Mongolian plateau^34^, which led to decreased surface wind speeds, as indicated in Fig. 4a and c. Compared with a meridional pattern, the noticeable decline of westerly maximum wind speed greater than 0.9 m/s is found since 2000 near the Taklimakan desert regions (40–50°N, 80–90°E) in China. However, there is only a slight positive trend in the meridional mean of the maximum wind speed in China sporadically since 1970 (Fig. 4b and d). Therefore, we note that a steady decrease in zonal maximum wind speed in the Northern Hemisphere was largely responsible for this recent decline in dust event occurrences.

To address this issue, the evolution of natural forcing parameters, including precipitation, potential evapotranspiration (PET), and aridity, are analyzed in Supplementary Fig. S2. At a regional scale, the increase in precipitation is much smaller across northern China than the increase in PET, which leads a decrease in the aridity index in the middle- and high-latitude areas. Therefore, based on the meridional averaged values of the aridity index, a significant expansion of drylands in China has occurred, especially in the semi-arid regions^36^. However, the results are inconsistent with the decrease in dust events over northern China, revealing that the significant changes in dust events are not controlled by dryland expansion due to enhanced land warming^36–38^. However, recent analyses have indicated that anthropogenic mineral dust events have occurred more frequently in semi-arid regions, which are more sensitive to human activities^29,39,40^.

In this study, six typical sites are selected to determine the contributions of natural and anthropogenic dust to the dust column burden. As shown in Supplementary Fig. S3, Lanzhou, located in the center of China, and Yinchuan, located 450 km away from Lanzhou, were both influenced by natural dust events from desert regions and local air pollution due to human activities. The other three sites including Beijing, Taiyuan, and Zhengzhou represent downwelling regions, which feature more anthropogenic air pollutants due to human activities. Erenhot is the only selected site close to the Gobi Desert region near the northern border of central Inner Mongolia, and it shows the highest dust storms frequency in the study area (see Supplementary Fig. S4f). The other five sites have experienced increased in blowing dust and floating dust since 1960, with the large switch occurring at the beginning of the 1980s.

To our knowledge, dust events are mainly related to dust storms outbreaks driven by natural forces. However, our data suggest that several dust events were produced due to land use, such as harvesting, plowing, overgrazing, cement production, construction, or road transportation. As shown in Supplementary Fig. S5, the evolutions of dust event frequency for both floating dust and blowing dust show significant decreasing trends, while the frequency of dust storms exhibits only slight variation at the typical sites. At Yinchuan and Lanzhou, blowing and floating dust have consistently decreased since the 1960s. At Erenhot, close to Gobi source regions, the floating dust decreased substantially, whereas blowing dust experienced a slight increase at the beginning of the 1980s. Zhengzhou, Beijing, and Taiyuan, which have the largest human populations, appeared to show a decreasing trend in blowing dust, likely due to the decreased wind speed in the eastern part of China.

**Figure 5:**
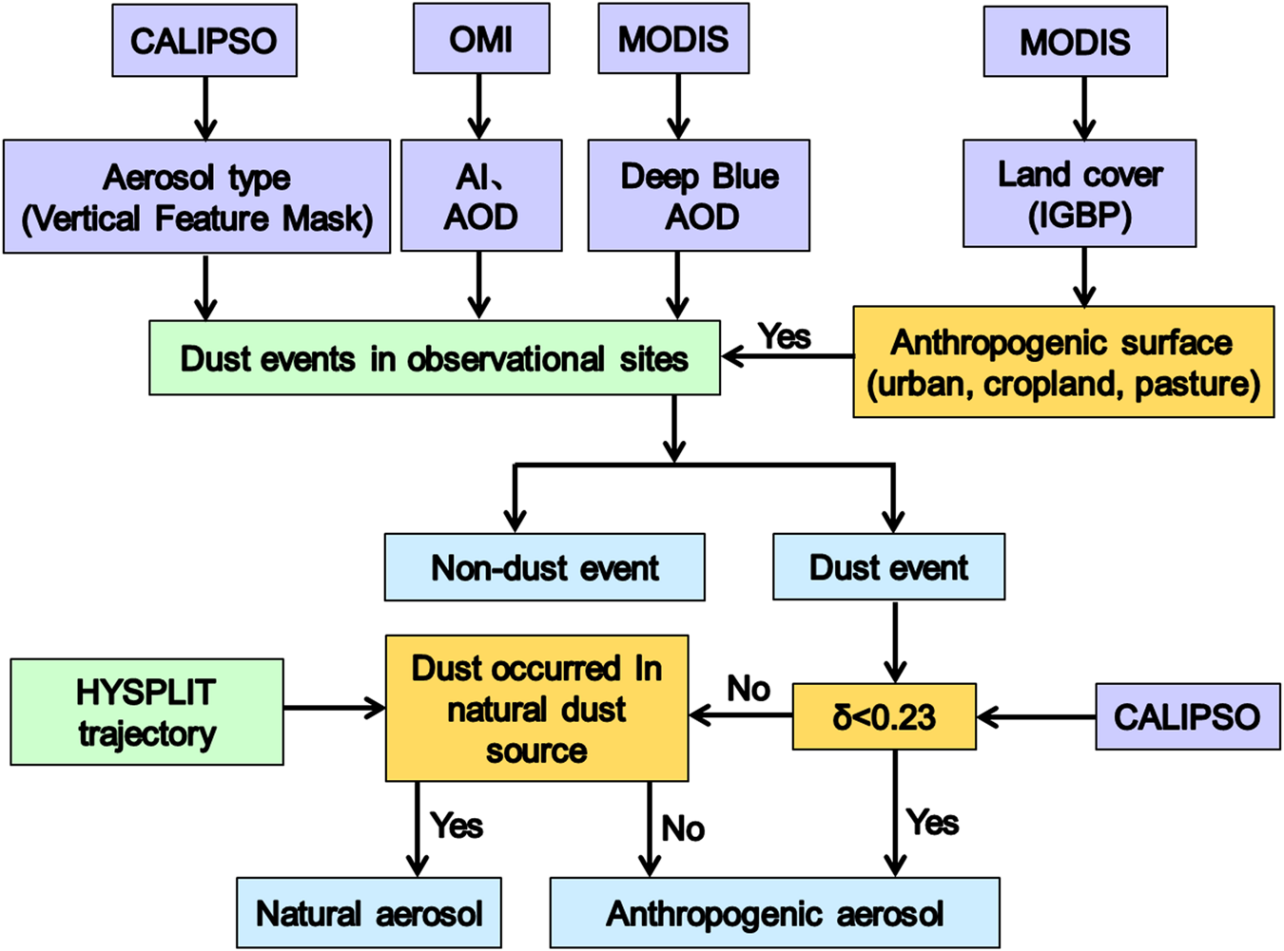
Flow chart of the definition of anthropogenic and natural dust detection by combing multi-satellite datasets, HYSPLIT trajectory, observational dust episodes, and the MODIS land cover dataset.

As shown in Fig. 5, the method for separating natural and anthropogenic dust is mainly based on the detection and identification methods described in ref.^26^. However, we also use the surface meteorological observations of dust events associated with air mass trajectories to reduce the uncertainty in the determination of whether the dust event originated from remote desert or local (urban) regions. To examine the results of this retrieval technique, two individual cases of anthropogenic and natural dust are shown in Supplementary Figs S6 and S7, respectively. As shown in Supplementary Fig. S6, a significant anthropogenic dust event occurred in eastern China. The 72-hour back trajectories originated from the southern regions, and no significant transport of dust aerosols occurred from the desert regions. Up to 70% percentage of the depolarization ratios calculated using Cloud-Aerosol Lidar and Infrared Pathfinder Satellite Observation (CALIPSO) datasets were lower than 0.23. However, based on ref.^26^, a large bias existed for natural dust that originated from desert regions. For instance, significant transport of natural dust is shown in Supplementary Fig. S7. However, 40% percentage of the depolarization ratios of this case are also generally lower than 0.23. This result is significantly inconsistent with the method developed in ref.^26^. Therefore, we indicated that the method in this study can detect anthropogenic dust more accurately and can reduce the large uncertainty associated with estimating the contribution of anthropogenic dust to total dust emissions.

**Figure 6:**
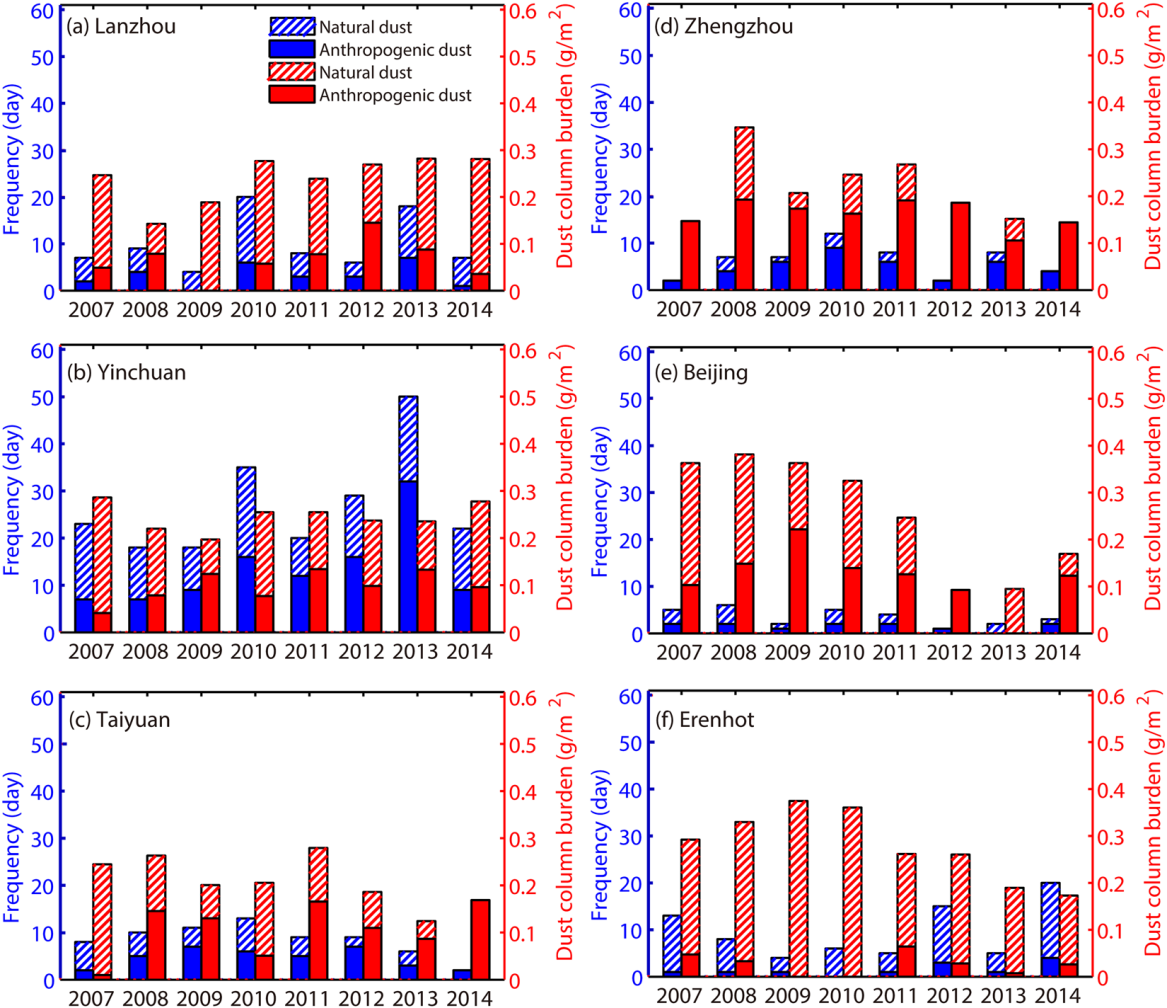
The frequency (Blue) and column burden (Red) of anthropogenic and natural dust episodes at typical observational sites during 2007–2014.

The lowest frequency of anthropogenic dust occurrences (<3 per year) is found in Erenhot (Fig. 6f), which features more natural dust events due to its proximity to the desert regions. In contrast, the highest frequency of total dust event occurrences is at Yinchuan due to the proximity of the two largest deserts and rapid industrialization (Fig. 6b). However, the percentage of anthropogenic dust to total dust occurrences at Yinchuan is lower than that at Taiyuan and Zhengzhou, which are situated in these regions most heavily polluted by human activities (Fig. 6c and d). Compared with the other regions, Beijing shows the lowest occurrences of both anthropogenic and natural dust events (Fig. 6e). There are two possible reasons for this phenomenon, with the more important being the significant reduction in wind speed caused by the three northern shelter forests, as this wind speed reduction decreases the frequency of dust events. For the selected sites, the average dust column burdens are 0.24 (Lanzhou), 0.25 (Yinchuan), 0.28 (Erenhot), 0.21 (Taiyuan), 0.21 (Zhengzhou) and 0.25 g·m^−2^ (Beijing). The annual anthropogenic dust column burdens show significantly increased trends from 2007 to 2011 in Yinchuan, Taiyuan, and Zhengzhou due to the local air pollution and higher population densities in eastern China^39^, but gradually reduce since 2011 due to the emission control of air pollution by the government^41^. The annual mean contributions of the anthropogenic dust column burden to the regional total emissions at these three sites are 39.9%, 52.8%, and 76.8%, respectively. Erenhot, located close to the southern border of the Gobi Desert (a natural dust source region), is dominated by natural dust sources and features the lowest anthropogenic dust contribution (9.2%). The mean contribution of anthropogenic during dust events to the annual dust column burden, as retrieved by multi-satellite remote sensing instruments in this study, ranges from 27% to 77% across eastern China, with a minimum value of less than 9% near the desert source regions.

### Conclusion and Discussion

Based on the MEEMD analysis, the spatiotemporal evolution of dust events shows only slight decreases in dust event occurrences for all types of dust events during 1960–1979 but large declines since 1980 in northern China. The noticeable decline of westerly maximum wind speed greater than 0.9 m/s is found since 2000 near the Taklimakan desert regions, which is largely responsible for this recent decline in dust event occurrences. The contribution of anthropogenic dust to the total dust emissions estimated by satellite retrievals from 2007 to 2014 is less than 9.2% near desert regions but as high as 76.8% across eastern China. We note that the misidentified rates of anthropogenic dust and natural dust using the detection technique are approximately 4.7% and 2.4%, respectively, thus representing a reduction in the source uncertainties associated with land cover data. We note that this study highlights the importance of improving the calculation of the contribution of natural dust and anthropogenic dust in terms of spatiotemporal allocation in order to further improve remote sensing techniques and reduce the uncertainties in model simulations, thereby enhancing our understanding of the climate impacts of anthropogenic mineral dust in the atmosphere.

### Data and Methods

The number of days per year on which dust events occurred at 1,754 observational sites (annual dust events >1) and the spatial distribution of the drylands are shown in Supplementary Fig. S1. The datasets of daily dust event occurrences for the period 1960–2014 derived from surface meteorological observations over mainland China provided by the National Meteorological Center of China. The monthly precipitation datasets from 1960 to 2008 used in this study were obtained from the National Centers for Environmental Prediction (NCEP) Climate Prediction Center (CPC), which cover the global land area with a spatial resolution of 0.5° × 0.5°. The aridity index is defined as the ratio of annual precipitation to annual PET and represents the degree of climatic dryness. The long-term average of this ratio is termed aridity (Ā). According to the World Atlas of Desertification, drylands are regions of Ā < 0.65 and can be further classified into hyper-arid (Ā < 0.05), arid (0.05 ≤ Ā < 0.2), semi-arid (0.2 ≤ Ā < 0.5), and dry subhumid (0.5 ≤ Ā < 0.65) subtypes. PET is calculated using the solar radiation, specific humidity, and wind speed reanalysis datasets from the Global Land Data Assimilation System (GLDAS) with a 0.5° × 0.5° latitude-longitude resolution starting from 1948^42^.

The current study relies on the Level 1 backscatter and depolarization ratio profiles associated with the Level 2 Vertical Feature Mask (VFM) products and 5-km Aerosol Profile Products using CALIPSO Cloud-Aerosol Lidar. The VFM product provides information about cloud and aerosol layer boundaries and positions^43,44^. In CALIPSO version 3 VFM data, the cloud aerosol discrimination (CAD) algorithm separates clouds and aerosols based on multidimensional histograms of scattering properties. In addition, Moderate resolution imaging spectroradiometer (MODIS) level 2 the Deep Blue aerosol optical depth (AOD) data at 550 nm with a spatial resolution of 10 × 10 km provided by the Terra platform was used in this study. The aerosol index (AI) measurements from the Ozone Monitoring Instrument (OMI) also provide valuable datasets on the distribution of light absorbing particles and has a spatial resolution of 13 × 24 km. The Collection 5.1 MODIS global land cover type product (MCD12C1) includes 17 different surface vegetation types and was developed by the International Geosphere–Biosphere Programme data (IGBP). This study uses three agricultural surface types: croplands, grasslands, and cropland mosaics. The Global Rural–Urban Mapping Project (GRUMP) v1 dataset with a spatial resolution of 500 m was also used to determine the extent of urban areas, representing one of the four anthropogenic dust source types. The Air Resources Laboratory (ARL) Hybrid Single-Particle Lagrangian Integrated Trajectory Model (HYSPLIT) was also used to locate the possible dust emission sources. A detailed dust detection algorithm between natural and anthropogenic dust is shown in Fig. 5.

The calculation of the dust column burden was performed as follows. First, we used the dust extinction coefficient through the parameter “Atmospheric Volume Description” to discriminate between aerosols and clouds in the CALIPSO Level 2 aerosol extinction profile products. Then, dust extinction coefficients with higher confidence levels (|CAD| ≥ 70) and quality control (QC) values (QC = 0 or QC = 1) were selected^45^. Then, dust optical depth (DOD, τ) was calculated by integrating the CAD and QC quality-controlled extinction coefficients of dust aerosols over the height of the dust layer. After calculating the global total DOD (τ_t_) and anthropogenic DOD (τ_a_) from the CALIPSO profile products between 2007 and 2014, we were able to calculate dust column burdens. The conversion from DOD (τ) to dust column mass burden (M) was calculated as follows^46^:

1 $${\rm{M}}=\frac{4}{3}\frac{{{\rm{\rho }}{\rm{r}}}_{{\rm{reff}}}}{{{\rm{Q}}}_{{\rm{ext}}}}{\rm{\tau }}=\frac{1}{{\rm{\varepsilon }}}{\rm{\tau }}$$

where r_reff_ is the dust effective radius, ρ is the density of dust, Q_ext_ is the dust extinction efficiency, and ε is the mass extinction efficiency. In this study, we follow the empirical values used by ref.^22^ and assume r_reff_ = 1.2 µm, ρ = 2600 kg. m^−3^, Q_ext_ = 2.5, ε = 0.6 m^2^. g^−1^, and τ is the dust optical depth derived from the CALIPSO retrievals.

Finally, the MEEMD technique for multi-dimensional data has been used in this study to separate the spatiotemporal varying trends from spatially non-uniform variability. The decomposition is based on the use of the ensemble empirical mode decomposition (EEMD) to slice the data in each grid and every dimension involved^47,48^. Briefly, EEMD is an adaptive one-dimensional data analysis method^49^ that can reflect the non-linear and non-stationary nature of climate data, as shown in Supplementary Fig. S5. For multi-dimensional temporal and spatial data in Fig. 2, EEMD is applied to a time series for each spatial location to obtain the corresponding intrinsic mode function of different time scales of dust event occurrences. A detailed description of the MEEMD method and the associated noise-assisted data analysis can be found in ref.^47^. The equation describing the EEMD method is as follows:

2 $${\rm{x}}({\rm{t}})=\sum _{{\rm{j}}={\rm{1}}}^{{\rm{n}}}{{\rm{C}}}_{{\rm{j}}}({\rm{t}})+{{\rm{R}}}_{{\rm{n}}}({\rm{t}})$$

where x(t) is a time series at a given location, which is decomposed into terms of adaptively obtained, amplitude/frequency-modulated oscillatory components C_j_(t) (j = 1, 2, ……, n) and a residual term R_n_.

## Electronic supplementary material

Dust storm

Blowing dust

Floating dust

Supplementary Information
