# Supplementary material for: Spatial and temporal evolution of natural and anthropogenic dust events over northern China
Source: Sci Rep. 2018 Feb 1;8:2141. doi: 10.1038/s41598-018-20382-5 (PMC5795005; doi:10.1038/s41598-018-20382-5)
Supplement: Supplementary file 4 — Supplementary Information [file 41598_2018_20382_MOESM4_ESM.pdf]

Spatial and temporal evolution of natural and anthropogenic dust events over northern China

Xin Wang, Jun Liu, Huizheng Che, Fei Ji, and Jingjing Liu

Supplementary Figures

The following figures show the number of observational sites at which dust events occurred (annual dust events >1) and the dryland regions of China (Fig. S1). The figures also show the evolution of the zonal and meridional mean precipitation, PET, aridity index (Ā) (Fig. S2) in China during the study period; selected observational sites across anthropogenic land cover regions (Fig. S3), the long-term frequency trends of dust episodes in Lanzhou, Yinchuan, Taiyuan, Zhengzhou, Beijing, and Erenhot for 1960-2014 (Fig. S4); and the EEMD decomposition of the frequencies of dust episodes at observational sites for 1960-2014 (Fig. S5). Additionally, two examples of anthropogenic and natural dust detection (Fig. S6 and S7) are provided. Furthermore, Table S1 lists the contribution of anthropogenic dust to the total atmospheric dust column burden for 2007-2014.

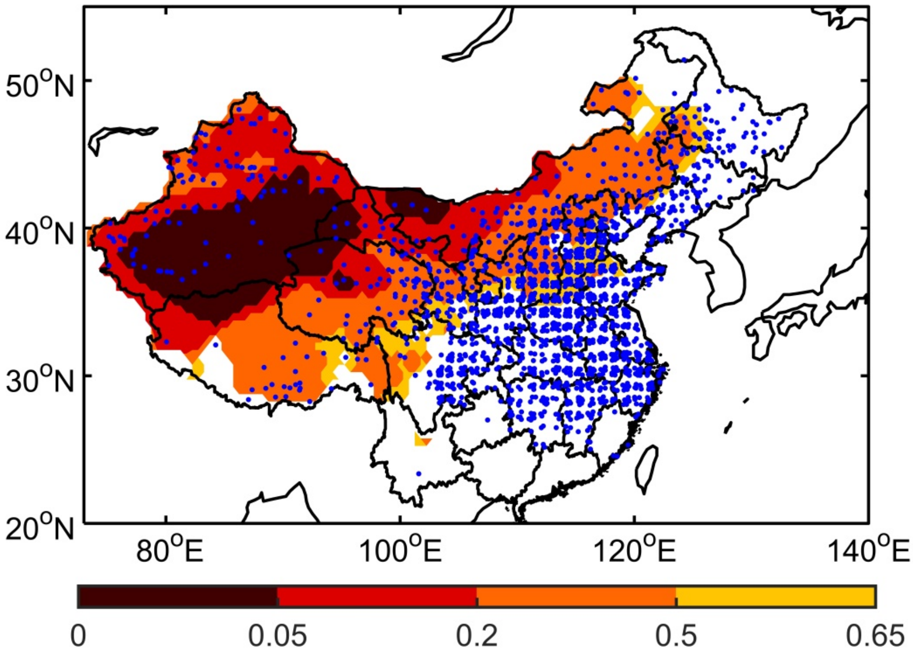

Figure S1. Locations of the 1,754 observational sites and the spatial distribution of dryland areas. The maps in the figure are generated using the MATLAB software (Version:R2016a(9.0.0.341360)&http://www.mathworks.com/products/matlab/?s_tid =srchtitle).

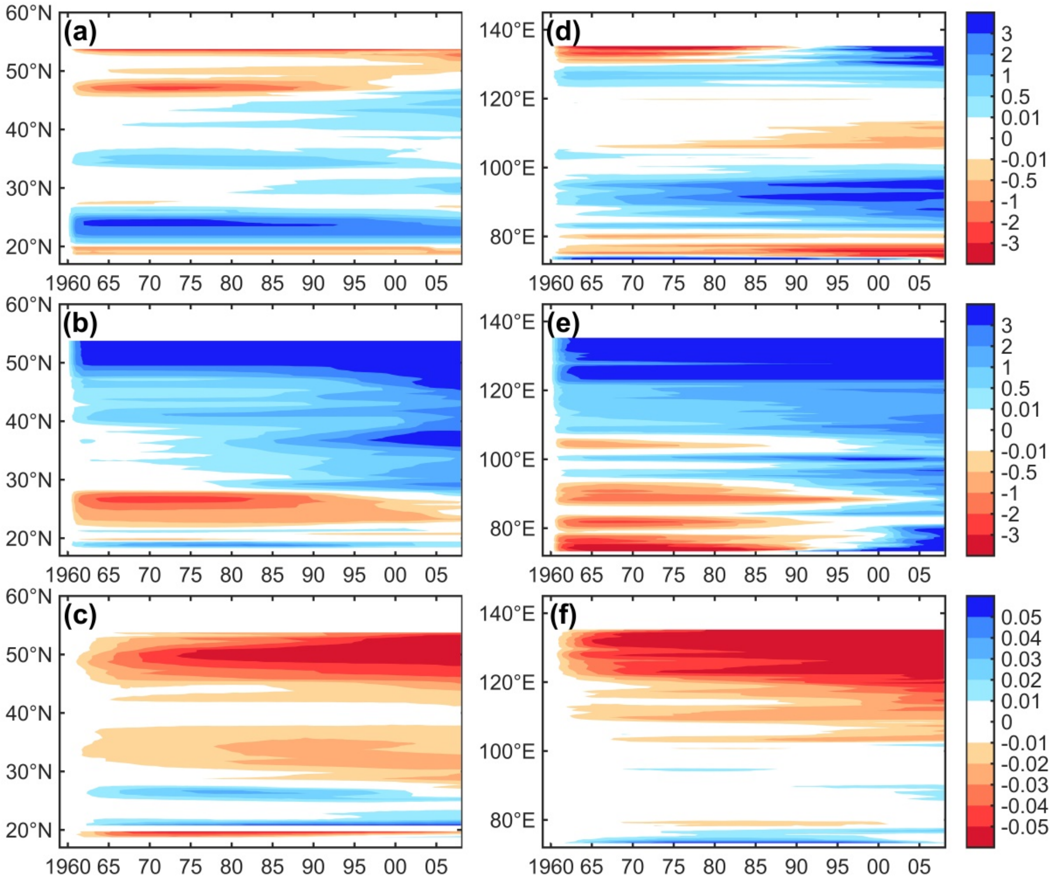

Figure S2. Evolution of the zonal mean trends in (a) precipitation, (b) PET, and (c) aridity index in China during the study period. From (d) to (f) Same as from (a) to (c) but for meridional mean.

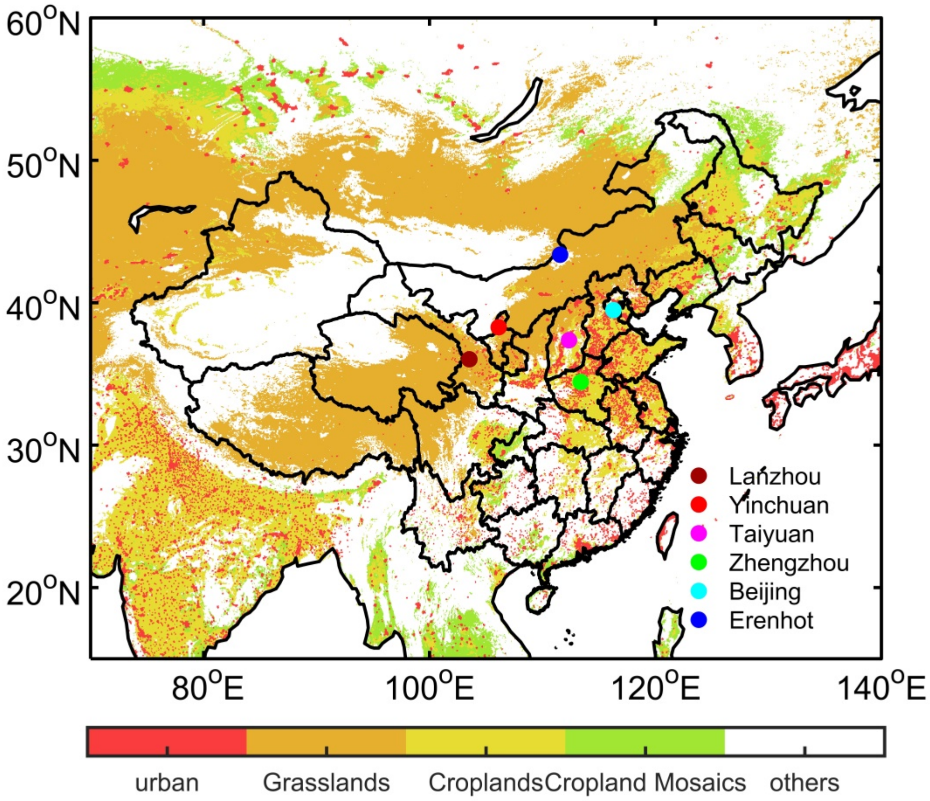

Figure S3. Selected observational sites across anthropogenic land cover regions (including urban, grasslands, cropland, and pastureland) retrieved by combing MODIS and GRUMP data. The map in the figure are generated using the MATLAB software (Version:R2016a(9.0.0.341360)&http://www.mathworks.com/products/matlab/?s_tid=srchtitle).

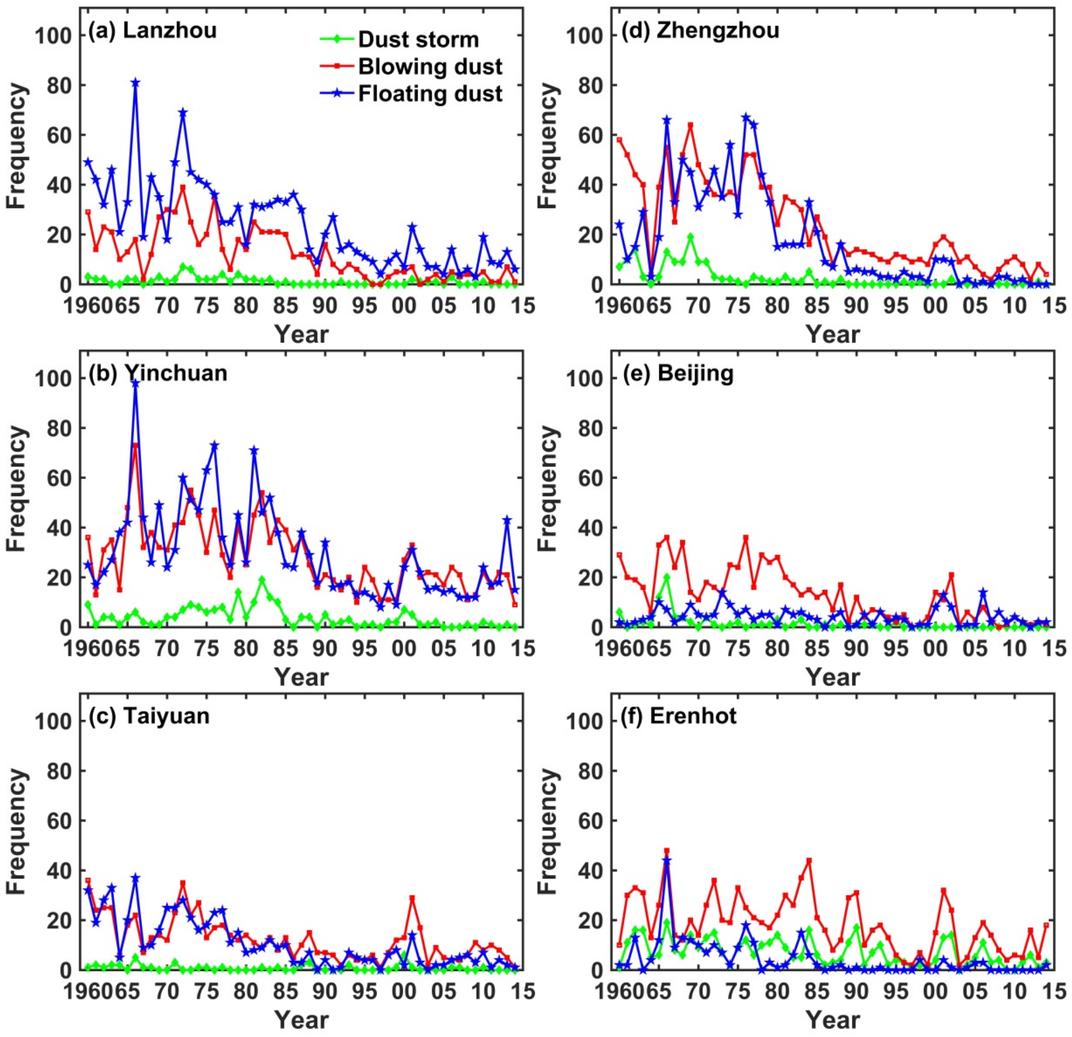

Figure S4. Frequencies of long-term trends in dust episodes at (a) Lanzhou, (b) Yinchuan, (c) Taiyuan, (d) Zhengzhou, (e) Beijing, and (f) Erenhot for 1960-2014.

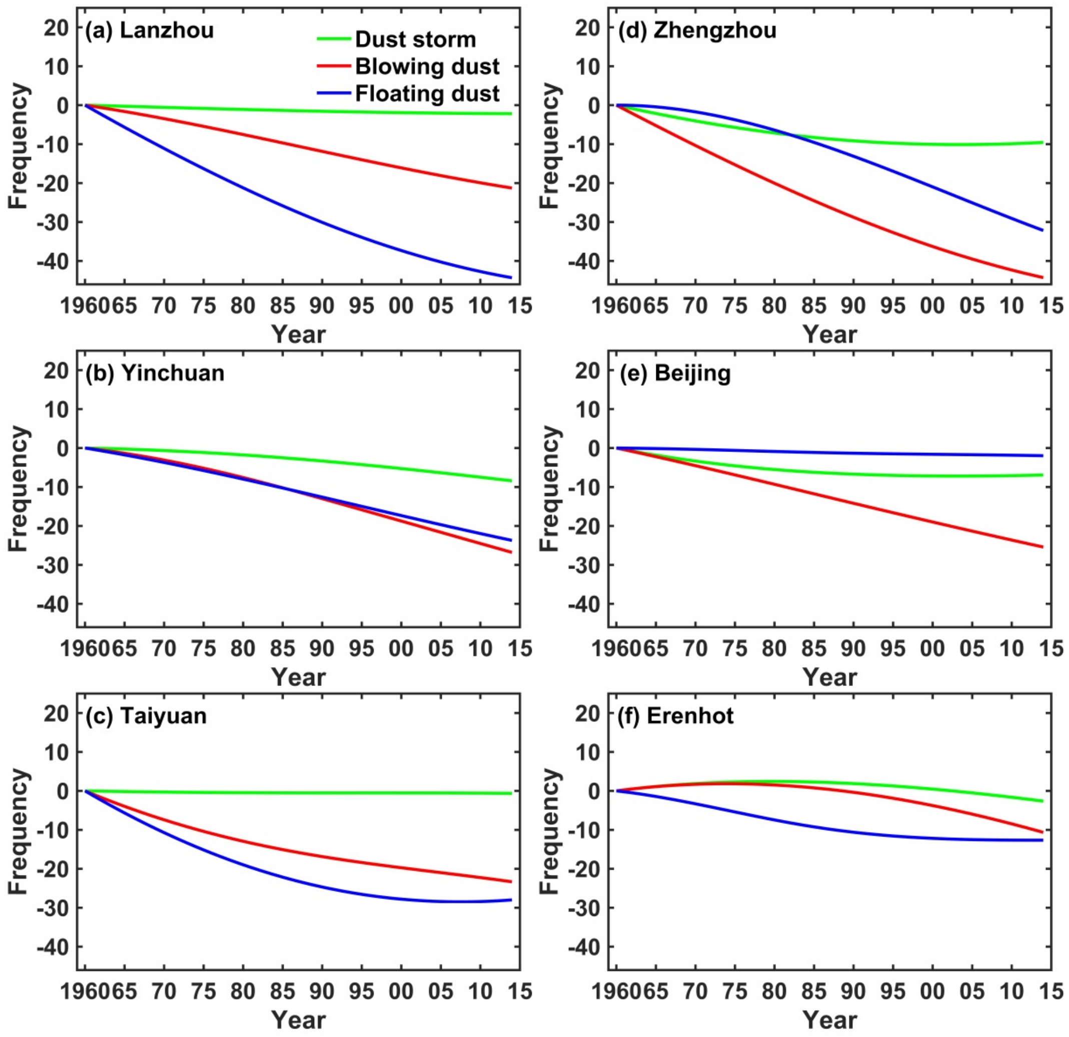

Figure S5. EEMD decomposition of the frequencies of dust episodes at observational sites for 1960-2014.

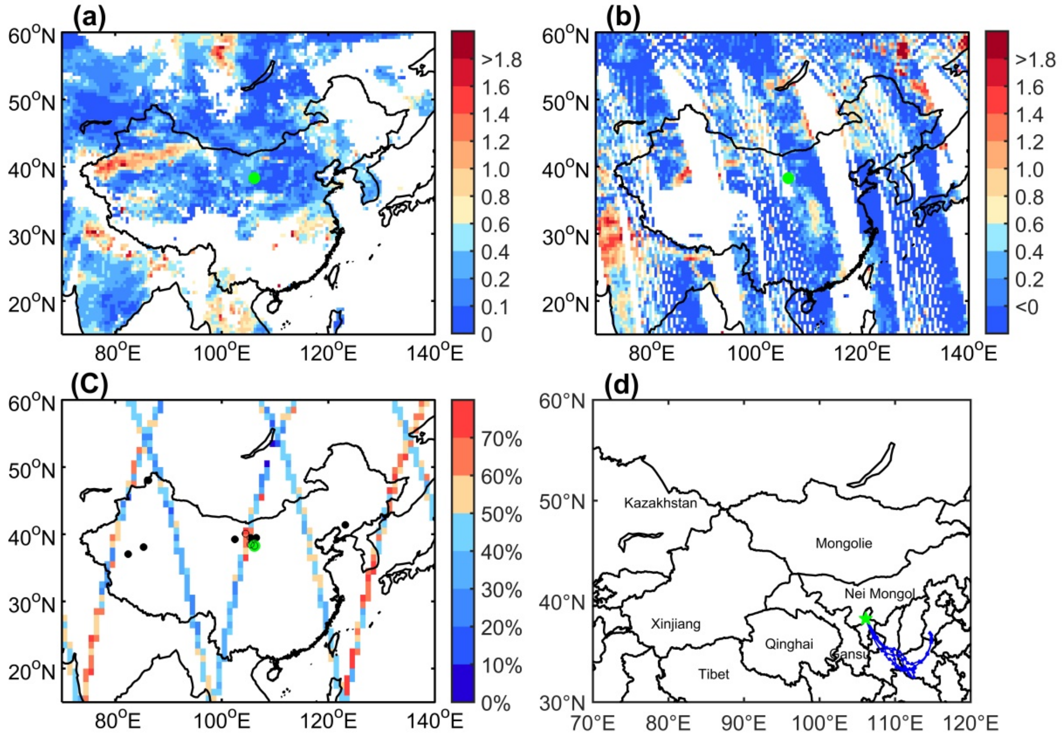

Figure S6. (a) Aerosol optical depth, (b) aerosol index, (c) depolarization ratio, and (d) back trajectory during an anthropogenic dust event. The maps in the figure are generated using the MATLAB software (Version: R2016a (9.0.0.341360)& http://www.mathworks.com/Products/matlab/?s_tid=srchtitle).

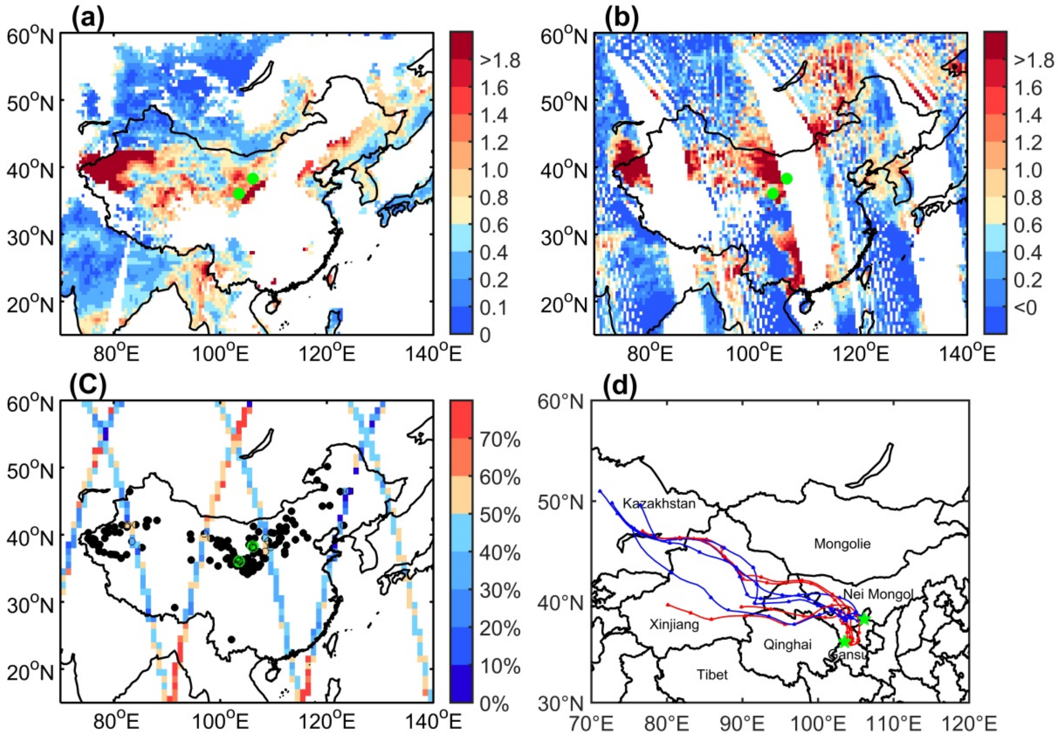

Figure S7. (a) Aerosol optical depth, (b) aerosol index, (c) depolarization ratio, and (d) back trajectory during a natural dust event. The maps in the figure are generated using the MATLAB software (Version: R2016a(9.0.0.341360)& http://www.mathworks.com/Products/matlab/?s_tid=srchtitle).

Table S1 **C**ontribution of anthropogenic dust to the total atmospheric dust column burden for 2007-2014.

**Sites**	**Total Dust**	**Anthropogenic dust**	**Percentage**
Lanzhou	0.24	0.07	27.6%
Yinchuan	0.25	0.10	39.9%
Taiyuan	0.21	0.11	52.8%
Zhengzhou	0.21	0.16	76.8%
Beijing	0.25	0.12	46.8%
Erenhot	0.28	0.03	9.2%
